# Graphene on SiC(0001) inspected by dynamic atomic force microscopy at room temperature

**DOI:** 10.3762/bjnano.6.93

**Published:** 2015-04-07

**Authors:** Mykola Telychko, Jan Berger, Zsolt Majzik, Pavel Jelínek, Martin Švec

**Affiliations:** 1 Institute of Physics, Academy of Sciences of the Czech Republic, Cukrovarnická 10, CZ-16200 Prague, Czech Republic; 2 Charles University, Faculty of Mathematics and Physics, V Holešovičkách 2, Praha 8, Czech Republic; 3 Department of Physical Electronics, Faculty of Nuclear Sciences and Physical Engineering, Czech Technical University in Prague, Břehová 7, CZ-11519 Prague, Czech Republic

**Keywords:** AFM, electron scattering, graphene, SiC, STM

## Abstract

We investigated single-layer graphene on SiC(0001) by atomic force and tunneling current microscopy, to separate the topographic and electronic contributions from the overall landscape. The analysis revealed that the roughness evaluated from the atomic force maps is very low, in accord with theoretical simulations. We also observed that characteristic electron scattering effects on graphene edges and defects are not accompanied by any out-of-plane relaxations of carbon atoms.

## Introduction

Graphene epitaxially grown on a substrate differs in many aspects from free-standing graphene or graphene exfoliated onto insulating surfaces. The influence of the substrate hinders applications of epitaxial graphene in the nanoelectronics [1]. The two main methods of epitaxial graphene growth are chemical vapor deposition (CVD) on metal surfaces [2] and annealing of silicon carbide (SiC) [3]. The large conductivity of metal substrates leaves graphene on metals as model-only systems. Alternatively, graphene on SiC(0001) is a more promising candidate for applications [4–5]. But its electronic behaviour is still strongly affected by intrinsic properties of the substrate and the morphology of the interface [6].

Understanding the interplay between atomic and electronic structure of graphene grown on SiC substrate is a crucial factor for device construction. In this respect structural properties of graphene were extensively studied by atomic force microscopy (AFM) [7–9] and scanning tunneling microscopy (STM) [10–12]. STM measurements of single-layer (SLG) as well as bi-layer graphene (BLG) grown epitaxially on 6H- or 4H-SiC(0001) show a characteristic 6
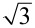
× 6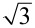 quasi-periodic corrugation (q-6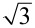 hereafter) [3,13–15]. It has not been clarified yet whether STM contrast on this surface has electronic or topographic origin. There is a lack of knowledge about the real physical corrugation of SLG and its contribution to the observed STM contrast, as it has already been successfully studied on graphene/Ir(111) [16–18]. However, it is well-known that the morphological modulations of the SLG arise from the interaction with the so-called buffer layer – a C-rich phase, which separates graphene from the SiC substrate [19–21].

Until now, the morphology of SLG on SiC(0001) has been studied by STM and AFM separately. Therefore, an investigation using the combined STM–AFM technique, that has simultaneous access to the electronic and topographic channels, is needed.

For the first time we bring experimental data, that can distinguish the topographic landscape from the local electronic structure of SLG on a 6H-SiC(0001) substrate. At room temperature we employed a combined STM and dynamic atomic force microscopy (dAFM) based on the Q-plus sensor working under UHV conditions. We observe the 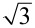
×
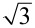
R30° (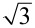) modulations, characteristic for the areas of graphene edges and point defects. Our results complement the previous findings about the origin of scattering at the defects and also serve for a comparison with properties of different kinds of epitaxial graphene.

## Experimental

The experiments were performed in an ultra-high vacuum (UHV) environment with a base pressure not exceeding 1 × 10 ^−10^ mbar. N-doped Si-face 6H-SiC(0001) wafers purchased from CREE Inc., were cut into 3 mm × 10 mm stripes and mounted onto sample holders constructed for a direct-current sample heating. After inserting into the UHV, the samples were degassed at 600 °C and annealed to 800–850 °C under a Si flux for 30 min, which resulted in a perfect Si type 3 × 3 reconstruction and typical terrace widths above 50 nm. As the source of the Si flux we used a Si wafer, which was annealed up to 1200 °C by passing the direct current. The deposition rate was 1 mL per 5 min. The temperature of the sample was measured using an optical pyrometer with spectral emissivity set to 0.6, operating at 1.6 μm wavelength, focused onto a spot on the sample with a diameter <2 mm. Buffer layer and graphene growth were achieved by further annealing of the sample up to 1150 °C, repeated in 10 min increments, until the desired coverage of graphene was achieved (about 2/3 of the surface) [13–14] and terrace widths reached 100 nm. All intermediate steps were monitored both by the low energy electron diffraction and STM. A custom-built quartz-tuning fork sensor was used for the measurements. It had a main resonance frequency of 51294 Hz, a quality factor above 1000 and an estimated stiffness of ≈3800 N·m^−1^ [22]. The contact to the tungsten tip was made of a thin golden wire in order to avoid crosstalk with the deflection signal from the tuning fork piezo [22–23]. The tip has been treated by annealing to 1200 °C in contact with a hot tungsten filament. The simultaneous current and frequency shift measurements were done in constant height mode. A very slow tunneling current feedback was applied for compensation of the sample tilt. The reason to use current as a feedback, as opposed to using the frequency shift (Δ*f*), is the possibility of doing measurements in the region of a negative frequency shift gradient (repulsive regime), even at room temperature, without enhanced risk of losing the tip apex. This approach is specifically chosen for the conditions when the graphene contrast provided by the Δ*f* is not giving atomic resolution in the attractive regime. Kelvin probe force measurements (KPFM) were also done in the constant-height mode, with a slow feedback between the measurement points, to compensate the tilt of the sample. KPFM parabola was measured by sweeping the bias voltage and measuring the Δ*f* value [24].

In order to estimate the SLG corrugation we carried out large scale total energy density functional theory (DFT) calculations. We used local-orbital FIREBALL code [25–26]. FIREBALL uses an optimized [27] spatially-confined pseudo-atomic numerical orbital basis set. In our case, an ss* basis set was used for the H atoms, a *sp* basis set for the Si and C atoms. The cutoff radii of the pseudo-atomic basis functions were as follows: *R*(H,*s*) = 4.5 a.u., *R*(H,s*) = 4.5 a.u., *R*(C,*s*) = 4.5 a.u, *R*(C,*s*) = 4.5 a.u., *R*(Si,*s*) = 6.0 a.u., *R*(Si,*p*) = 6.00 a.u. Local density approximation is used for the exchange correlation functional. Our atomic models of the SLG-SiC(0001) surface consisted of a slab of 1648 atoms, 10 atomic layers thick (including the buffer and SLG layer) with an additional passivating hydrogen layer on the underside. The lateral size of the supercell was 6
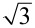
× 6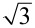. The calculations were restricted to the Γ point of the first surface Brillouin zone. The bottom Si atomic layer as well as the hydrogen layer were kept fixed during the geometry optimization while all other atoms were allowed to relax freely into their equilibrium positions. The criterion for terminating the relaxation was that maximal forces on free atoms had to be below 0.05 eV/Å and the change of total energy between subsequent iterations had to be smaller than 10^−4^ eV per unit cell.

## Results

**Figure 1:**
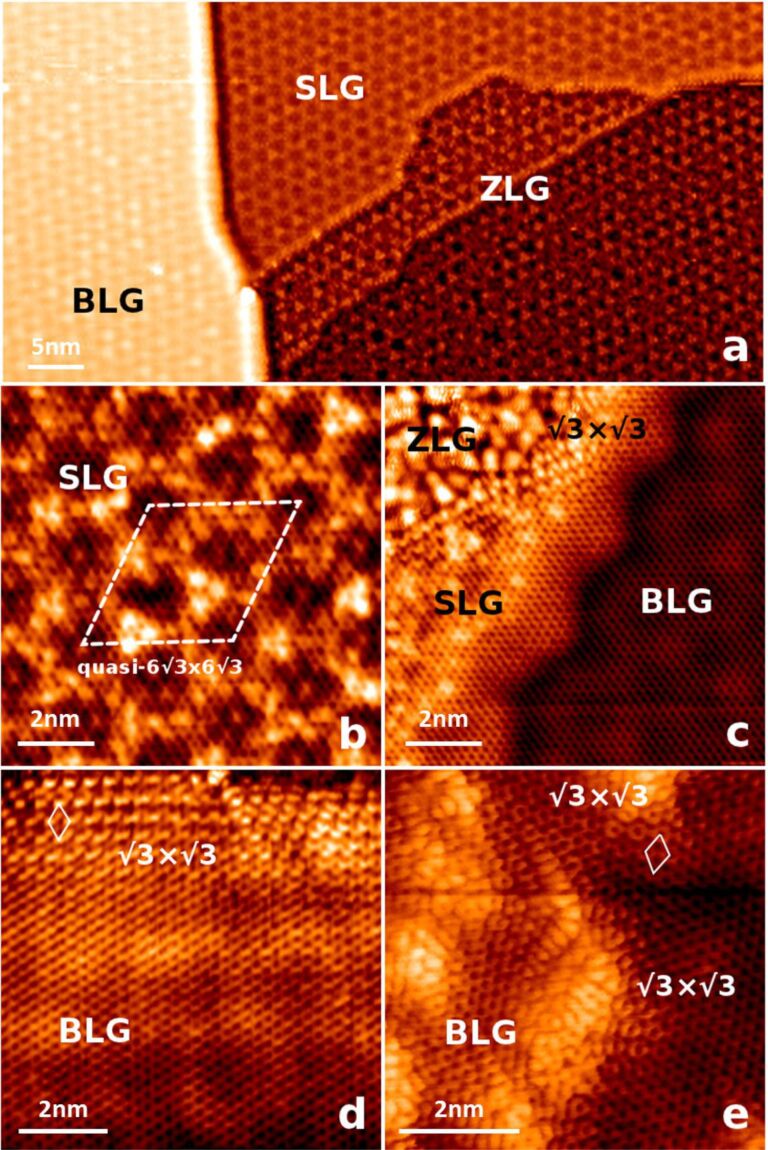
Constant-current STM images taken over an incomplete graphene layer grown on the 6H-SiC(0001) (color online). (a) 67 × 33 nm^2^, *V*_bias_ = −1.5 V, *I* = 0.15 nA; overview of an area containing the buffer layer (ZLG) and single- and bi-layer graphene (SLG and BLG). (b) 10 × 10 nm^2^, *V*_bias_ = 0.5 V, *I* = 0.18 nA; subsurface features bonds of the buffer layer visible through a sheet of graphene, the characteristic 6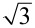 quasiperiodicity is indicated. (c) 10 × 10 nm^2^, *V_bias_* = 0.5 V, *I* = 0.18 nA; example of a characteristic 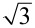
×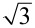 rippling of SLG near to an armchair edge, step overgrown by graphene without any extra rippling, (d) 8.5 × 8.5 nm^2^, *V*_bias_ = 0.2 V, *I* = 0.16 nA; rippling at a boundary between SLG and BLG graphene, (e) 6.4 × 6.4 nm^2^, *V*_bias_ = −0.2 V, *I* = 0.16 nA; 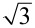
×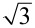 near to vacancies created by ion etching.

Figure 1 shows a compilation of STM images taken over an incomplete layer of graphene grown on 6H-SiC(0001). Figure 1a shows the main features of the surface morphology, terraces divided by steps of various heights, areas covered with SLG and BLG and areas of buffer layer. All of them have a common pattern – the quasiperiodic (q-6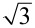) modulation corresponding to the 6
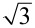
× 6
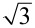
R30° quasiperiodic structure detected by low-energy electron diffraction [3,14]. Figure 1b shows a detail of the SLG, measured at a bias voltage 0.5 V, with a stable tip that provides a good resolution, allowing the detection of buffer layer features. These are responsible for the characteristic modulation of SLG. The roughness measured by the STM at voltages below 0.5 V has a typical value of 21 pm RMS and peak-to-peak value of 60 pm. Figures Figure 1c,d show rippled boundaries of graphene. The edges are rippled, regardless whether it is a BLG or SLG boundary, zigzag or armchair termination of the graphene domain. In a closer inspection of the armchair-type termination (FigureFigure 1d), it can be identified locally as a 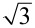 periodicity. This characteristic feature has been observed before, not only near to the graphene boundaries, but also around single-point defects in graphene and graphite [3,13,28–29] and adsorbates [30–32]. We also recorded a prominent example of this effect at ion-etched graphene – see Figure 1e. This effect is generally explained by intervalley scattering of electrons in graphene [33]. In contrast, in a special case where the graphene overgrows a step (see Figure 1c), there is no visible rippling.

**Figure 2:**
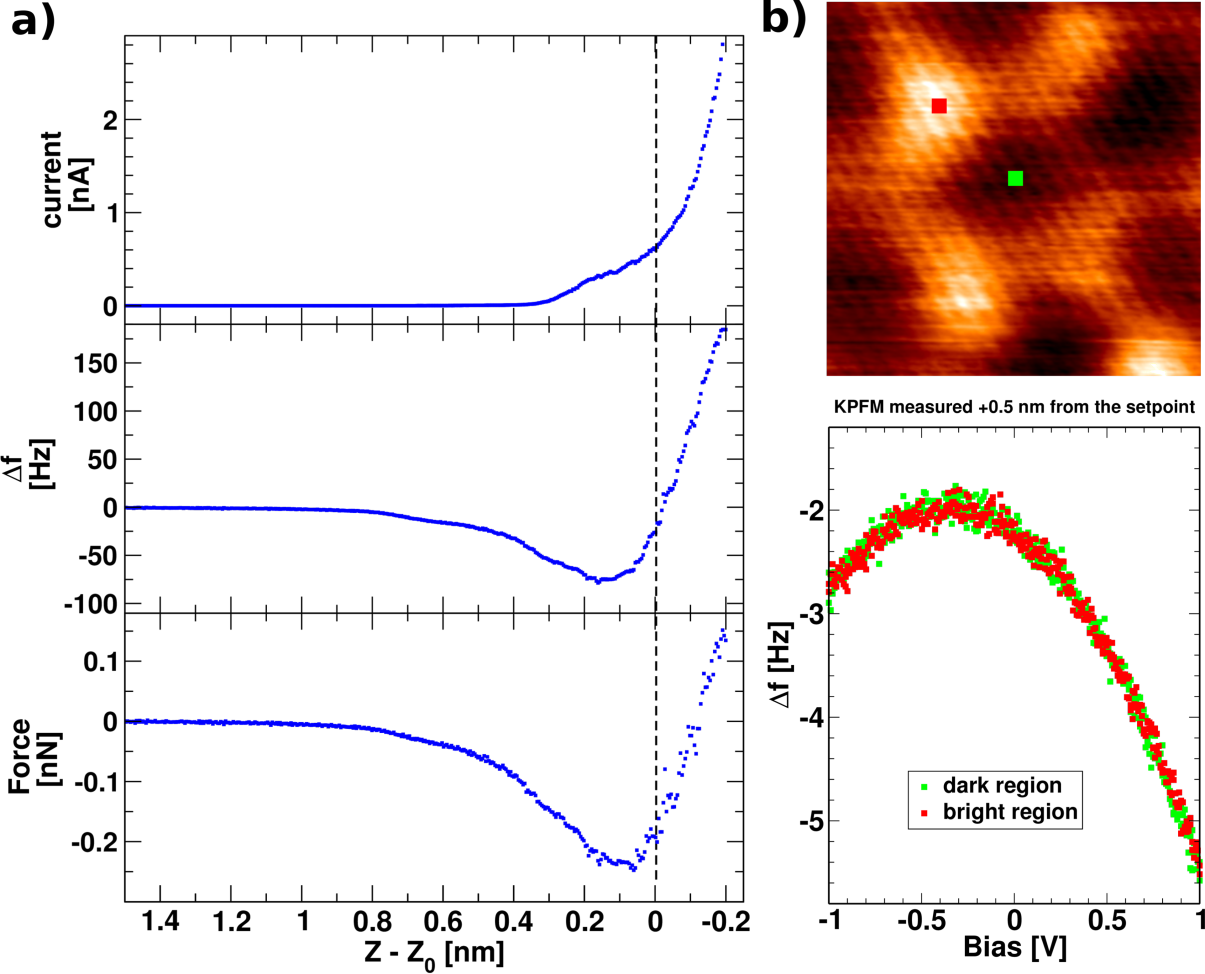
(Color online) (a) Spectroscopy curves of average tunneling current <*I*_t_>, Δ*f* and force *F* vs the tip–sample distance on graphene/SiC(0001), taken with a bias voltage of 100 mV, just before imaging. The value of *Z* = 0 corresponds to the tip–sample separation at <*I*_t_> setpoint of 0.46 nA. (b) Example measurements of the local potential at bright and dark regions of the q-(6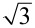) modulation visible in the Δ*f* map, indicating undetectable charge transfer.

The set of curves in Figure 2a shows local spectroscopy taken just before the imaging above the single-layer graphene with the tuning-fork-based dAFM sensor, oscillating at amplitudes of 150 pm. The Δ*f*, time-averaged current (<*I*_t_>) are measured vs the tip–sample relative distance (*Z*) during an approach and retraction of the tip. Voltage is kept at 100 mV. The <*I*_t_> grows exponentially with *Z* and the Δ*f* has a minimum near to −80 Hz. The value of *Z* = 0 corresponds to the tip–sample separation at <*I*_t_> setpoint of 0.46 nA. In the region of the set point, the Δ*f* is rising at a rate of (0.98 ± 0.04) Hz/pm, as determined by the least-square fitting. Force (*F*) is calculated from the Δ*f* signal, using the approach proposed by Sader et al. [34]. Maximum attractive force amounts roughly to −0.3 nN.

To identify any charge transfer effects within the q-6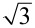 structure of graphene a Kelvin-probe measurement [24] has been performed on the bright and dark areas (highs and lows of the q-6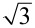 modulation) in the Δ*f* image, at 0.5 nm from the setpoint, further from the sample. In Figure 2b, the two corresponding parabolas show no difference neither in the shape nor in their maxima. The contact potential between the tip and the sample corresponds to maxima in the parabolas and is located at −0.32 eV.

**Figure 3:**
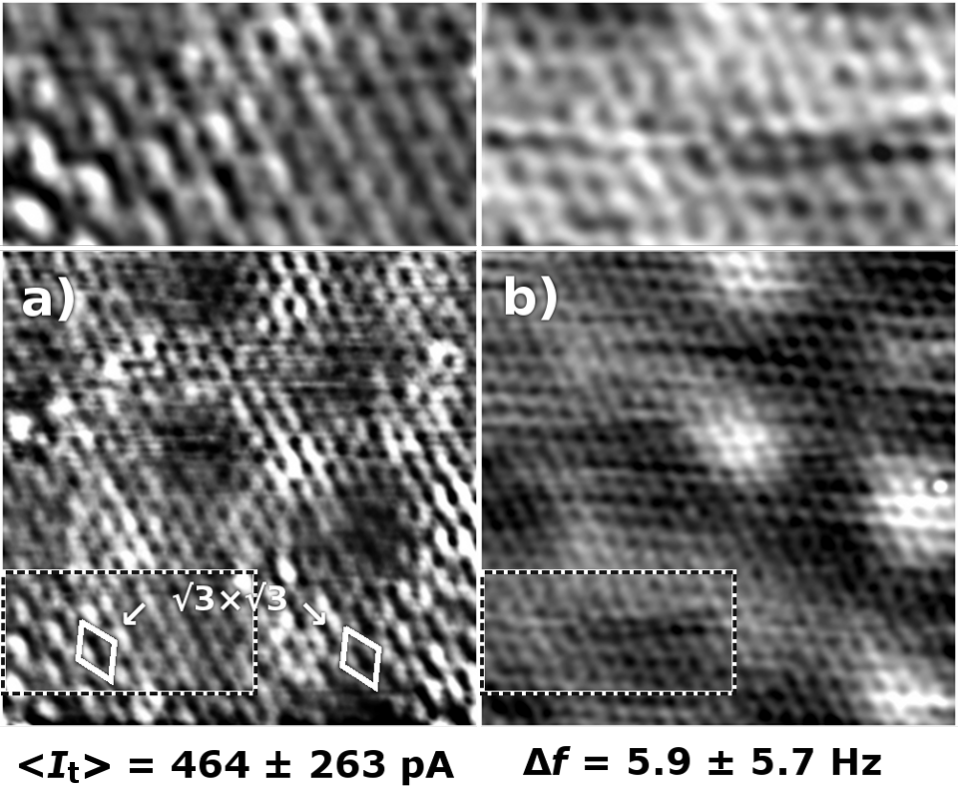
(a) The average tunneling current <*I*_t_> maps (*V*_bias_ = 100 mV, *I* = 0.46 nA, 5.5 nm × 7.1 nm and (b) frequency shift Δ*f* acquired on the same area of graphene/SiC(0001). The q-6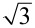 modulation is apparent in both channels, whereas the 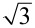
×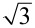 modulation due to scattering on armchair boundary is detected only in the <*I*_t_> map. The insets on the top show zoomed images of a region that has a 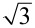
×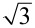 modulation in the <*I*_t_> map. The mean value and a standard deviation (RMS) are given for each measured quantity beneath each of the images.

In Figure 3, the Δ*f* and <*I*_t_> maps are presented for the same area of the sample, taken in a constant height regime with a slow feedback setpoint at 0.46 nA, producing a tip–sample separation close to the *Z* = 0 in Figure 2a. The <*I*_t_> map shows an image resembling the STM topography in Figure 1b, however with a slightly sharper contrast, which is a common aspect of current images taken in the constant-height regime [35]. It shows the characteristic graphene honeycomb structure modulated by the q-6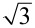 periodicity. The Δ*f* signal shows the honeycomb structure and the q-6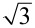 modulation as well. In this case, the slow feedback control of the tip–sample distance allows us to stay in the repulsive regime, achieving atomic resolution. This would be otherwise possible only at very stable conditions, e.g., at low temperatures. The lower part of the <*I*_t_> image in Figure 3 (also zoomed in the inset) possesses features arising from armchair graphene boundaries. The boundaries residing in the left and right lower corners, produce characteristic 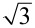 ripples on graphene visible in the <*I*_t_> channel. In contrast, the Δ*f* image shows only a flat honeycomb structure of graphene.

## Discussion

**Figure 4:**
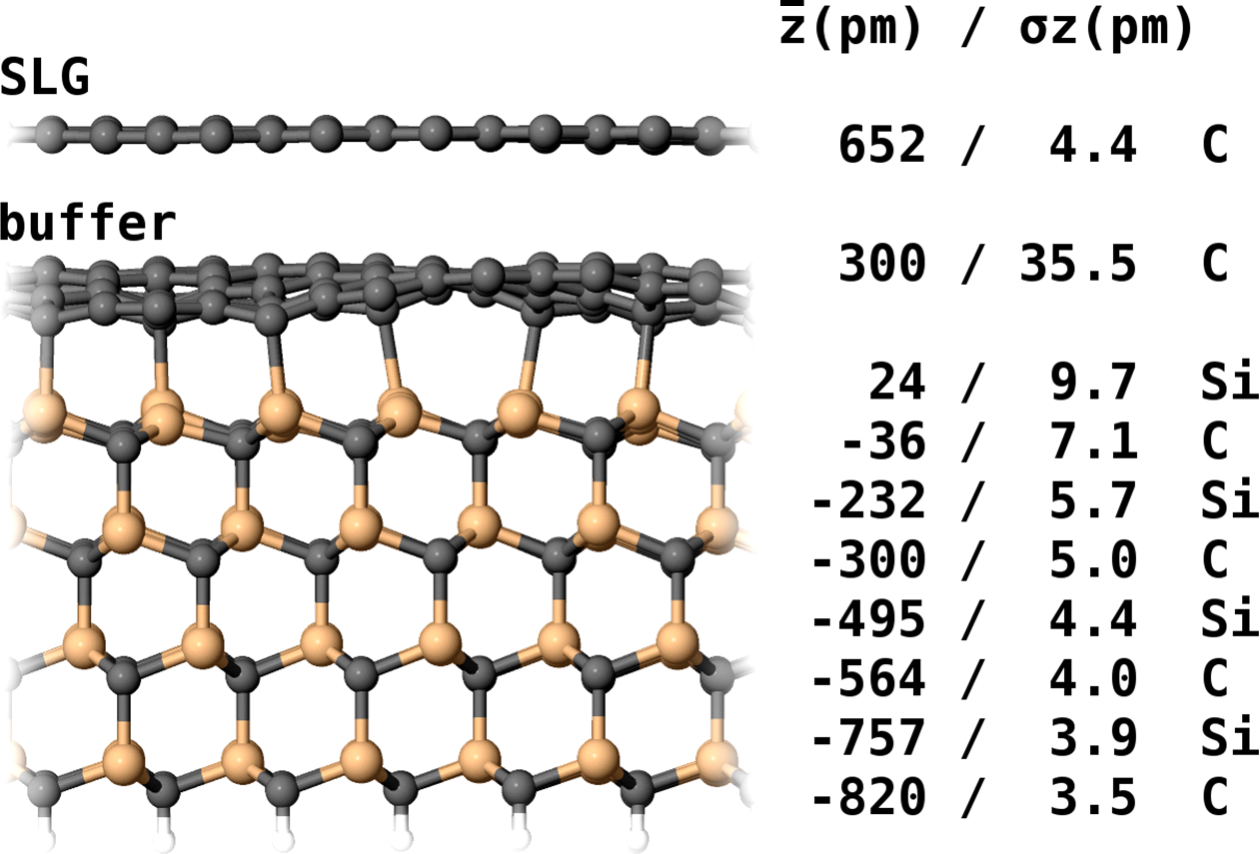
The fully relaxed theoretical model of SLG on the buffer layer, that is using the 6
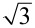
× 6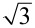 unit cell. The z values in the table correspond to z-positions of C and Si atomic layers in the model, σz is a standard deviation of atom positions. The standard deviation of the C atom z-positions in the SLG layer is 4.4 pm.

The experimental results reveal that the q-6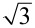 modulation of graphene in STM has, apart from a significant electronic contribution, a topographic component. It is a question how strong is the topographic effect and whether it can be evaluated from the semi-constant height data. Neglecting the effect of the slow feedback and by using the Δ*f*(*Z*) curve from Figure 2a, we estimated the *Z* height modulation of graphene from the Δ*f* images in Figure 3. Considering that the image is taken in the negative Δ*f* region, which has a of 1.0 Hz/pm, we use the Δ*f* roughness corresponding to the q-6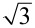 modulation (5.7 Hz RMS), to obtain an average value of about 6 pm RMS, corresponding to 16 pm corrugation (peak-to-peak). This is significantly lower than the roughness obtained by standard topographic mode of STM, pointing out the dominant electronic contribution of the buffer layer to the STM topography of SLG. In our fully relaxed theoretical model of SLG on the buffer layer (Figure 4), which is using the 6
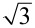
× 6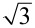 unit cell, the standard deviation of the C atom z-positions in the SLG layer is 4.4 pm (12.4 pm peak-to-peak), which corresponds well to the experimental estimation and is comparable to a similar calculation in [36]. The value is much smaller than the corrugation obtained for graphene/Ru(0001) and graphene/Ir(111) that are both claimed to be in the range of 100 pm (corresponding to 35 pm RMS) [37–39].

Most remarkable is the 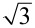 pattern (shown in the inset of Figure 3a) emerging due to the scattering at the armchair graphene boundaries. We do not observe any out-of-plane relaxation of the carbon atoms in the honeycomb structure resolved by dAFM (in the inset of Figure 3b). This is a direct evidence that the 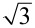 has a purely electronic origin. That is an expected behaviour since the effect has been attributed to scattering of Dirac electrons on graphene sublattices [31]. It also demonstrates that the graphene layer in SLG is weakly bound to the buffer layer through van der Waals interactions. This finding of no atomic relaxations can be extrapolated for the single-point defects and other graphene disruptions for graphene on SiC(0001), since they produce the same type of scattering and do not interact strongly with the substrate. In this entire observation, we can exclude any influence of the so-called phantom force [40], as the Δ*f* signal has no hints of the 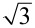.

Considering that the electronic contribution is the major factor that affects the <*I*_t_> maps, we can also deduce that these maps taken at low bias reflect a variation of local conductivity. Moreover, the Kelvin parabola measurements do not detect any significant contact potential difference between the dark and bright protrusions of the q-6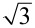 modulation. It can be understood as a negligible workfunction difference between these investigated areas.

## Conclusion

In summary, we successfully probed the epitaxial graphene on SiC(0001) by a combined STM and dAFM technique, gathering extended information on its atomic structure near defects. By atomic force microscopy, we detected a topographical corrugation of the q-6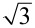 with an average value of 6 pm RMS (16 pm peak-to-peak). We can conclude that variation at this scale detected by previous STM measurements is caused mainly by electronic contributions arising from the buffer layer. In the case of the characteristic 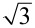 rippling of graphene, which occurs due to presence of defects or boundaries, any relaxations of carbon atoms perpendicular to the surface can be excluded. These results are consistent with recent findings and show the single-layer graphene on SiC(0001) as a morphologically very flat substrate.

## Acknowledgements

The research was funded by GACR grant no.14-02079S, GACR grant no. EXCELENCE 14-374527G.
